# Curcumin Attenuates *Escherichia coli*-Induced Enteritis and Macrophage Inflammation in Broilers

**DOI:** 10.3390/ani16182942

**Published:** 2026-09-19

**Authors:** Haiping Xu, Yantian Tang, Siyu Zhang, Guosheng Wang

**Affiliations:** 1 School of Food & Pharmaceutical Science and Technology, Guangzhou College of Technology and Business, Guangzhou 510850, China; xuhaiping@gzgs.edu.cn; 2 Heyuan Branch, Guangdong Laboratory for Lingnan Modern Agriculture, Heyuan 517000, China; 3 Fujian Key Laboratory of Animal Genetics and Breeding, Institute of Animal Husbandry and Veterinary Medicine, Fujian Academy of Agricultural Sciences, Fuzhou 350013, China; 4 Heyuan Customs Comprehensive Technical Service Center, Heyuan 517000, China; chwgsh@163.com

**Keywords:** curcumin, *Escherichia coli*, macrophage, gut inflammation, p38 MAPK

## Abstract

*Escherichia coli* (*E. coli*)-induced enteritis impairs intestinal barrier function, immune homeostasis, and growth performance in broilers. This study investigated whether dietary curcumin alleviates *E. coli*-induced intestinal injury by modulating macrophage-mediated inflammatory responses through the p38 MAPK signaling pathway. Broilers were fed either a basal or curcumin-supplemented diet and challenged with *E. coli*. Growth performance, intestinal inflammatory cytokines, morphology, barrier-related gene expression, goblet cell density, and ileal macrophage responses were evaluated. Lipopolysaccharide (LPS)-stimulated HD11 cells were used to examine the underlying mechanism. Curcumin supplementation increased body weight, average daily feed intake, and average daily gain in *E. coli*-challenged broilers, without significantly affecting the feed conversion ratio. Curcumin also reduced jejunal and ileal IL-1β, IL-6, and TNF-α levels; improved villus morphology and the villus height-to-crypt depth ratio; and upregulated *CLDN1*, *OCLN*, *ZO-1*, and *MUC2* expression. In addition, curcumin restored goblet cell density and ileal macrophage abundance while suppressing macrophage-derived pro-inflammatory cytokine expression. In HD11 cells, curcumin inhibited LPS-induced inflammatory cytokine expression and secretion and reduced p38 MAPK phosphorylation, whereas pharmacological activation of p38 partially reversed these effects. In conclusion, dietary curcumin alleviated *E. coli*-induced enteritis in broilers by suppressing intestinal inflammation, preserving intestinal barrier function, and modulating macrophage-mediated immune responses. In vitro findings further suggest that inhibition of p38 MAPK signaling may contribute to the anti-inflammatory effects of curcumin on chicken macrophages.

## Simple Summary

*Escherichia coli* infection can damage the intestine of broiler chickens, trigger excessive inflammation, and reduce growth performance. This study examined whether dietary curcumin could protect broilers against this intestinal injury. Curcumin supplementation improved body weight, feed intake, and weight gain, reduced intestinal inflammatory signals, and helped maintain the structure and protective function of the intestinal lining. It also restored mucus-producing goblet cells and improved the balance and inflammatory activity of intestinal macrophages, which are immune cells involved in controlling infection and tissue damage. Cell experiments further showed that curcumin reduced inflammation by suppressing a cellular signaling pathway known as p38 mitogen-activated protein kinase. These findings indicate that curcumin may be a useful natural feed additive for reducing intestinal inflammation and improving gut health in broiler production, thereby supporting animal health and sustainable poultry farming.

## 1. Introduction

The intestinal tract of chicks is not fully developed and is susceptible to environmental stress, dietary mycotoxins, and pathogenic infections, which may subsequently induce intestinal inflammation [1,2]. Intestinal inflammation can disrupt the intestinal mucosal barrier, impair epithelial function and nutrient absorption, and disturb immune homeostasis, thereby compromising growth performance and health in broilers [3]. *Escherichia coli* (*E. coli*) is an important opportunistic pathogen associated with intestinal infection and inflammatory responses in broilers [4]. *E. coli* infection can promote the release of intestinal inflammatory cytokines, damage the intestinal mucosal barrier, and exacerbate histopathological lesions in intestinal tissues. Therefore, exploring safe and effective nutritional strategies to alleviate *E. coli*-induced enteritis in broilers is essential for improving intestinal health and production efficiency.

Curcumin, a natural polyphenol derived from the rhizome of *Curcuma longa* L., exhibits anti-inflammatory, antibacterial, and immunomodulatory activities [5,6]. In recent years, curcumin has attracted considerable attention for its potential application in poultry nutrition and intestinal health management. Curcumin can maintain relatively high concentrations in the stomach and small intestine, suggesting that the gastrointestinal tract may be an important target site for its local biological effects [7]. Previous studies have shown that curcumin can improve intestinal digestion, absorption, and mucosal barrier function [8,9]. In broilers, dietary supplementation with an appropriate level of curcumin has also been reported to enhance antioxidant capacity and immune function and to upregulate the expression of tight junction proteins, including *CLDN1*, *OCLN*, and *ZO-1*, thereby maintaining intestinal barrier integrity [10]. These findings suggest that curcumin may protect against intestinal inflammation in broilers by modulating intestinal barrier function and immune-inflammatory responses.

Intestinal macrophages are critical innate immune cells that maintain intestinal immune homeostasis and orchestrate inflammatory response. Upon recognition of pathogen-associated molecular patterns, these cells are rapidly activated and secrete pro-inflammatory cytokines, including IL-6, IL-1β, and TNF-α. [11,12]. However, persistent or excessive macrophage-mediated inflammation can exacerbate intestinal injury, compromise barrier integrity, and perpetuate a vicious cycle of inflammation [13,14]. Thus, modulation of intestinal macrophage-mediated inflammatory responses represents a promising strategy for alleviating enteritis in broilers. The MAPK signaling cascade, which includes the ERK, JNK, and p38 MAPK pathways, is a key intracellular network involved in the regulation of inflammatory responses, with the p38 MAPK pathway playing a pivotal role in macrophage activation induced by pathogenic infection and inflammatory stimuli [15]. Activation of p38 MAPK promotes the expression of downstream transcription factors and inflammatory cytokines, driving the production of mediators such as IL-6, IL-1β, and TNF-α, while inhibition of excessive p38 MAPK activation has been shown to attenuate macrophage-mediated inflammation and ameliorate inflammatory tissue damage [16]. Collectively, these findings suggest that the p38 MAPK signaling pathway may serve as an important molecular target through which curcumin regulates intestinal macrophage-mediated inflammatory responses in broilers.

In this study, an *E. coli*-induced enteritis model in broilers was used to investigate the regulatory effects of curcumin on intestinal inflammatory injury and intestinal macrophage-mediated inflammatory responses. Furthermore, the potential involvement of the p38 MAPK signaling pathway in this process was examined. This study aimed to elucidate the immunomodulatory mechanism by which curcumin alleviates enteritis in broilers and to provide a theoretical basis for the application of curcumin in the regulation of intestinal health in broilers.

## 2. Materials and Methods

### 2.1. Experimental Design

Healthy 1-day-old Qingyuan broiler breeder chicks were purchased from Zhaoqing Yingnongxing Breeding Development Co., Ltd. (Zhaoqing, China). Qingyuan chickens are a representative Chinese yellow-feathered broiler breed. The chicks were randomly assigned to 4 groups using the random-number method, with 6 replicates per group and 6 chicks per replicate. The treatments were as follows: NC, fed a basal diet; Cur, fed the basal diet supplemented with 200 mg/kg curcumin; *E. coli*, fed the basal diet and challenged with *E. coli*; and CE, fed the basal diet supplemented with 200 mg/kg curcumin and challenged with *E. coli*. The challenged and non-challenged groups were housed separately to avoid cross-infection. All chicks were reared in floor pens bedded with approximately 5 cm of wood shavings. During the experiment, chicks had ad libitum access to feed and water. The basal diet was a corn–wheat–soybean meal-based diet formulated to meet the nutritional requirements of Chinese yellow-feathered broilers. The composition and nutrient levels of the basal diet are shown in Table S1. The *E. coli* challenge was performed at 08:00 on days 14, 17, and 20 of the experiment. Chicks in the challenged groups were orally gavaged with 2 mL of *E. coli K88* suspension at a concentration of 1 × 10^9^ CFU/mL. The sample size and challenge concentration were determined based on previous studies using comparable broiler challenge models and experimental conditions [6]. The *E. coli K88* standard strain was obtained from the Guangdong Microbial Culture Collection Center. Curcumin, with a purity greater than 98%, was provided by Kehu Biotechnology Co., Ltd. (Guangzhou, China).

### 2.2. Sample Collection

On day 21, all chicks were weighed to evaluate growth performance. Blood samples were collected from the wing vein and allowed to clot at 4 °C for 20 min, followed by centrifugation at 3000× *g* for 10 min. The serum was collected and stored at −20 °C for subsequent immune index analysis. After blood collection, the chicks were electrically stunned and euthanized by exsanguination. Jejunal and ileal tissue samples were rapidly collected. A portion of the intestinal tissues was frozen in liquid nitrogen and stored at −80 °C for subsequent analysis of cytokine levels and related gene expression. Another portion was fixed in 4% paraformaldehyde for histological analysis. Fresh ileal tissues were collected into prechilled sterile centrifuge tubes and temporarily stored at 4 °C for flow cytometric analysis.

### 2.3. Cell Culture

Chicken macrophage cell line HD11 was kindly provided by Professor Guobin Chang (Key Laboratory of Animal Genetics and Breeding and Molecular Design of Jiangsu Province, Yangzhou University, Yangzhou, China). HD11 cells were cultured in RPMI 1640 medium (Gibco, Carlsbad, CA, USA) supplemented with 10% fetal bovine serum, 100 U/mL penicillin, and 100 μg/mL streptomycin (Gibco, Carlsbad, CA, USA). Cells were maintained at 39 °C in a humidified incubator containing 5% CO_2_. For curcumin treatment, cells were preincubated with 10 μM Cur for 6 h, then cultured in the presence of 1000 ng/mL LPS (Sigma, St. Louis, MO, USA) for 3 to 12 h [17]. For p38 MAPK activator treatment [18], HD11 cells were pretreated with 100 ng/mL Anisomycin (Beyotime, Shanghai, China) for 5 min, then co-cultured with Cur and LPS.

### 2.4. Quantitative Real-Time PCR Analysis (qPCR)

Total RNA was extracted from jejunal and ileal tissues using RNAiso Plus reagent (Takara, Dalian, China) according to the manufacturer’s instructions. After RNA quality and concentration were assessed, reverse transcription was performed using the Evo M-MLV RT Premix for qPCR kit (Accurate, Changsha, China). Quantitative real-time PCR was performed using ChamQ Universal SYBR qPCR Master Mix (Vazyme, Nanjing, China). The qPCR reaction system had a total volume of 10 μL and contained 5 μL of SYBR Mix, 1 μL of cDNA, 0.5 μL each of forward and reverse primers, and 3 μL of ddH_2_O. Reactions were performed using a Bio-Rad real-time PCR detection system (Bio-Rad, Hercules, CA, USA). The amplification program was as follows: initial denaturation at 95 °C for 5 min followed by 40 cycles of denaturation at 95 °C for 30 s, annealing at 60 °C for 30 s, and extension at 72 °C for 30 s, with a final extension at 72 °C for 5 min. Primer amplification efficiencies were validated using standard curves, and β-actin showed stable expression across treatments. All primers used in this study were synthesized by Tsingke Biotechnology Co., Ltd. (Guangzhou, China), and the primer sequences are listed in Table S2. Relative gene expression levels were calculated using the 2^−ΔΔCt^ method, with *β-actin* used as the internal reference gene for normalization.

### 2.5. Western Blot Analysis

Protein samples were separated by 10% sodium dodecyl sulfate–polyacrylamide gel electrophoresis, then transferred onto nitrocellulose membranes. The membranes were blocked with 5% fat-free milk at room temperature for 2 h, followed by overnight incubation with the corresponding primary antibodies at 4 °C. After washing, the membranes were incubated with horseradish peroxidase-conjugated secondary antibodies at room temperature for 2 h. Protein bands were visualized using an enhanced chemiluminescence detection system and imaged with a FluorChem E system (Bio-Techne, Minneapolis, MN, USA). Specific antibodies against total p38 (#bs-28027R, 1:1000), phosphorylated p38 (p-p38, #bs-2210R, 1:1000) and β-actin (#bs-0061R, 1:1000) and secondary antibodies (#bs-40295G, 1:500) were purchased from Bioss Inc. (Beijing, China).

### 2.6. Enzyme-Linked Immunosorbent Assay (ELISA)

Commercial chicken-specific ELISA kits were used to determine the protein concentrations of IL-1β, IL-6 and TNF-α in serum and intestinal tissues according to the manufacturer’s instructions. The ELISA kits were purchased from Jiangsu Meimian Industrial Co., Ltd. (Yancheng, China). All samples were assayed in duplicate according to the kit instructions, and cytokine concentrations were calculated from standard curves.

### 2.7. Flow Cytometric Analysis of Ileal Macrophages

Ileal tissues were minced and pre-digested in 5 mL of PBS at 37 °C with shaking at 200 rpm for 20 min. After washing with PBS, the tissue fragments were mechanically disrupted at 40 Hz for 20 s and subsequently digested in 1 mL of PBS containing 0.25% trypsin (#25200056, Gibco, Grand Island, NY, USA) at 37 °C and 200 rpm for 10 min. The cell suspension was filtered through a 70 μm cell strainer and centrifuged at 500× *g* for 5 min. The cell pellet was resuspended in PBS, and viable cells were counted and adjusted to 1 × 10^6^ cells per sample. Cells were then stained with FITC-conjugated anti-CD45 (#8270-02) and PE-conjugated anti-M0 (#8420-09) antibodies (SouthernBiotech, Birmingham, AL, USA) at 4 °C in the dark for 30 min. After washing with PBS, cells were analyzed by FACSCalibur flow (BD Biosciences, Franklin Lakes, NJ, USA).

### 2.8. Histological Analysis

Jejunal and ileal tissues were fixed in 4% paraformaldehyde for 24 h, dehydrated through a graded ethanol series, cleared in xylene, and embedded in paraffin. Paraffin sections were cut at a thickness of 3 μm. Sections were stained with hematoxylin and eosin (H&E) to evaluate intestinal morphology. Alcian blue–periodic acid–Schiff staining (AB-PAS) was used to determine the number of goblet cells. After staining, images were captured using a light microscope (Leica, Wetzlar, Germany). Three sections were prepared for each broiler, and six fields were randomly selected from each section for imaging and analysis. Measurements from these fields were averaged to obtain a single value for each broiler. Villus height and crypt depth were measured using Image-Pro Plus software (v6.0), and the number of goblet cells per 100 μm of villus length was calculated.

### 2.9. Statistical Analysis

All data were analyzed using GraphPad Prism software (v10.1.2). Data are presented as the mean ± standard deviation (mean ± SD). Normality and homogeneity of variance were assessed before ANOVA. In vivo data were analyzed using two-way ANOVA with curcumin supplementation and *E. coli* challenge as fixed factors, followed by simple-effects analysis. When a significant interaction was detected, simple-effects analysis was performed with Šídák’s multiple-comparisons test. In vitro Cur/LPS experiments were analyzed by two-way ANOVA where applicable, whereas anisomycin experiments were analyzed by one-way ANOVA followed by Duncan’s multiple range test. A value of *p* < 0.05 was considered statistically significant.

Differences among groups were analyzed by one-way analysis of variance followed by Duncan’s multiple range test. A value of * *p* < 0.05 was considered statistically significant, whereas ** *p* < 0.01 and *** *p* < 0.001 were considered highly significant.

## 3. Results

### 3.1. Curcumin Improves Growth Performance and Attenuates Intestinal Inflammation in E. coli-Challenged Broilers

**Figure 1:**
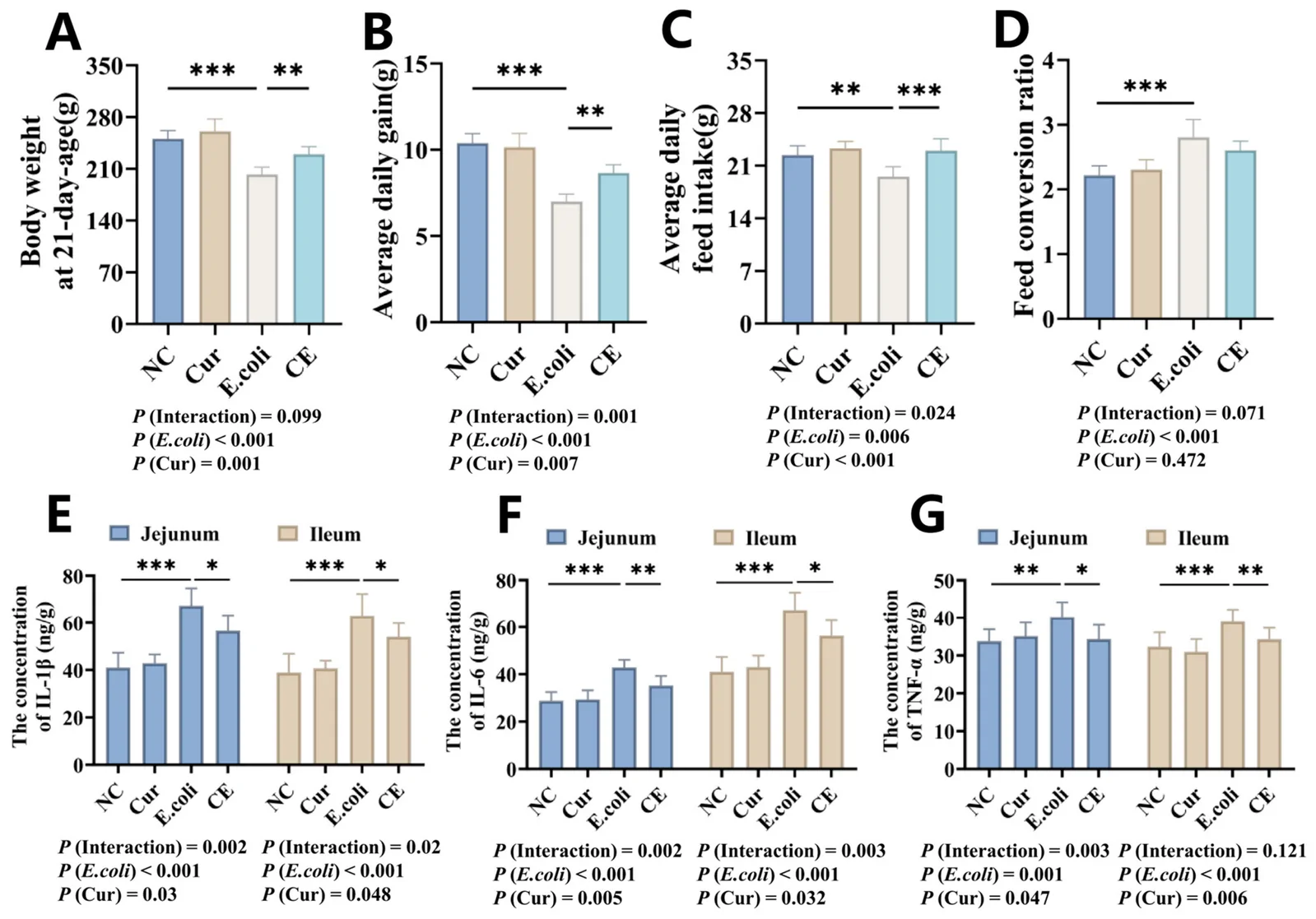
Curcumin improves growth performance and attenuates intestinal inflammation in *E. coli*-challenged broilers. (**A**) Body weight on day 21. (**B**) Average daily feed intake. (**C**) Average daily gain. (**D**) Feed conversion ratio. (**E**–**G**) The concentrations of TNF-α, IL-6, and IL-1β in the jejunum and ileum. NC, basal diet; Cur, basal diet supplemented with curcumin; *E. coli*, basal diet with *E. coli* challenge; CE, curcumin-supplemented diet with *E. coli* challenge. *n* = 6 (6 broilers per replicate). Data are presented as mean ± SD. * *p* < 0.05, ** *p* < 0.01, and *** *p* < 0.001.

The growth performance and intestinal inflammation indicators of curcumin-supplemented chicks after *E. coli* infection were evaluated. The results showed that the *E. coli* group had significantly reduced body weight (*p* < 0.001), average daily feed intake (ADFI, *p* < 0.01), and average daily gain (ADG, *p* < 0.001) and increased feed conversion ratio (FCR, *p* < 0.001) compared to the NC group, and compared with the *E. coli* group, the CE group showed increased body weight (*p* < 0.01), ADFI (*p* < 0.001) and ADG (*p* < 0.01), but no significant change in FCR was observed (Figure 1A–D). Regarding intestinal inflammatory cytokine, the *E. coli* group exhibited significantly increased levels of IL-1β (*p* < 0.001), IL-6 (*p* < 0.001), and TNF-α (*p* < 0.01) in the jejunum and ileum compared with the NC group, and the CE group showed reduced levels of IL-1β (*p* < 0.05), IL-6 (*p* < 0.05), and TNF-α (*p* < 0.05) in the jejunum and ileum compared with the *E. coli* group (Figure 1E–G). These results indicate that dietary curcumin supplementation significantly alleviated the *E. coli* challenge-induced intestinal inflammation and reductions in body weight, ADFI, and ADG but had no significant effect on FCR.

### 3.2. Curcumin Improves Intestinal Morphology and Restores Barrier Function in E. coli-Challenged Broilers

**Figure 2:**
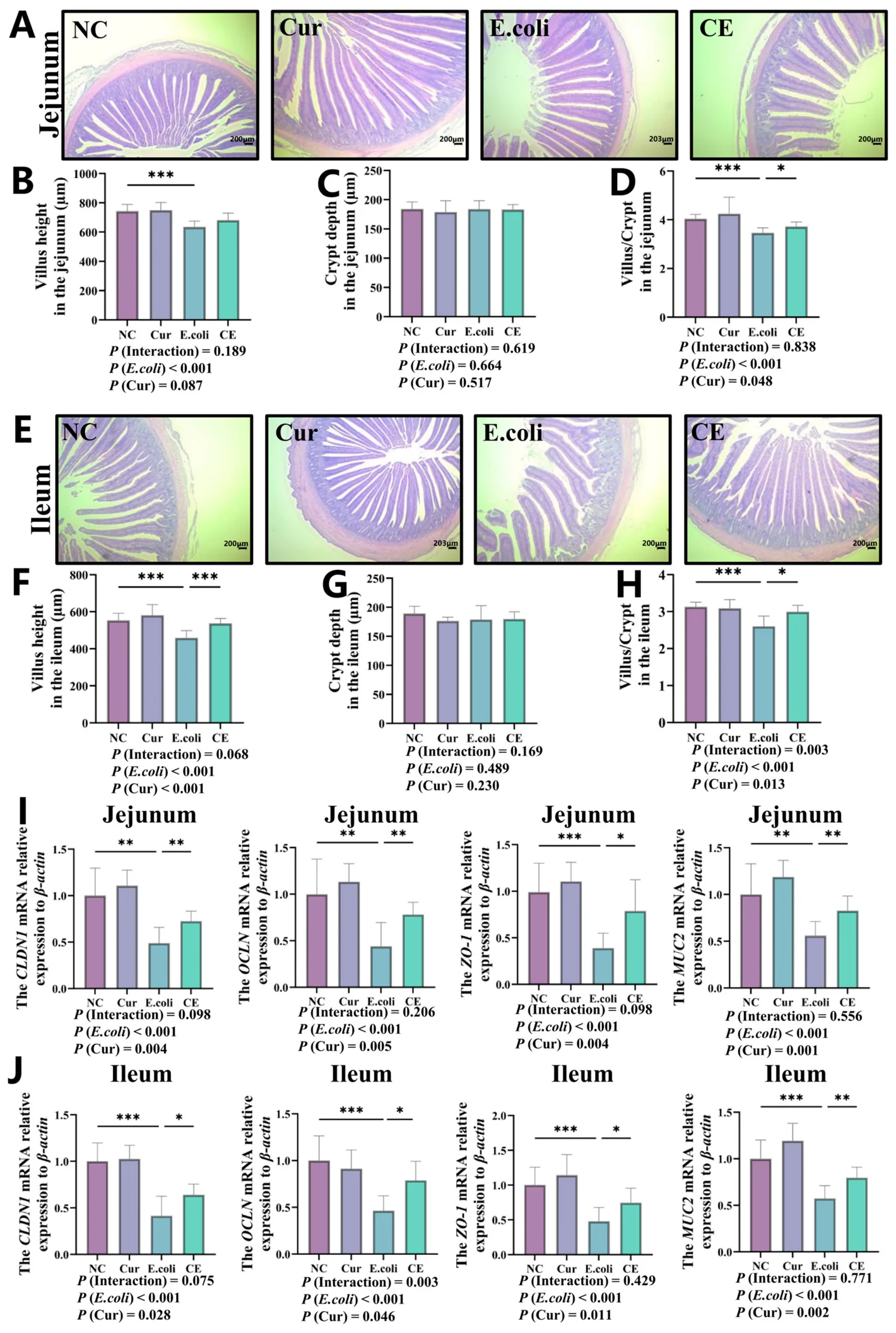
Curcumin improves intestinal morphology and restores barrier function in *E. coli*-challenged broilers. (**A**) Representative H&E-stained sections of the jejunum. (**B**–**D**) Jejunal villus height, crypt depth, and villus height-to-crypt depth ratio. (**E**) Representative H&E-stained sections of the ileum. (**F**–**H**) Ileal villus height, crypt depth, and villus height-to-crypt depth ratio. (**I**,**J**) Relative mRNA expression of intestinal barrier-related genes *CLDN1*, *OCLN*, *ZO-1*, and *MUC2* in the jejunum and ileum, respectively. NC, basal diet; Cur, basal diet supplemented with curcumin; *E. coli*, basal diet with *E. coli* challenge; CE, curcumin-supplemented diet with *E. coli* challenge. *n* = 6 (6 broilers per replicate). Data are presented as mean ± SD. * *p* < 0.05, ** *p* < 0.01, and *** *p* < 0.001.

Intestinal morphology and barrier function are important indicators in evaluating intestinal health and resistance to pathogen invasion. In this study, H&E staining was used to examine the morphology of jejunal and ileal tissues, and qPCR was performed to determine the mRNA expression levels of intestinal barrier-related genes. H&E results showed that the *E. coli* group had significantly decreased villus height (*p <* 0.001) and villus height-to-crypt depth (V/C) ratio (*p <* 0.001) in the jejunum compared with the NC group, and the jejunal V/C ratio (*p <* 0.05) was significantly increased in the CE group compared with the *E. coli* group (Figure 2A–D). In the ileum, the *E. coli* group also showed significant decreases in villus height (*p <* 0.001) and V/C ratio (*p <* 0.001), whereas the CE group exhibited significantly increased ileal villus height (*p <* 0.001) and villus height-to-crypt depth ratio (*p <* 0.05) compared with the *E. coli* group (Figure 2E–H). The mRNA expression levels of intestinal barrier-related genes in the jejunum and ileum were further analyzed by qPCR. Compared with the NC group, the *E. coli* group showed significantly downregulated mRNA expression of *CLDN1* (*p <* 0.01), *OCLN* (*p <* 0.01), *ZO-1* (*p <* 0.001), and *MUC2* (*p <* 0.01) in both the jejunum and ileum, and compared with the *E. coli* group, the CE group showed significantly increased expression levels of these genes (Figure 2I–J, *p <* 0.05). These findings suggest that curcumin effectively alleviated *E. coli*-induced impairment of intestinal barrier function.

### 3.3. Curcumin Increases Goblet Cell Density and Suppresses Ileal Macrophage Inflammation in E. coli-Challenged Broilers

**Figure 3:**
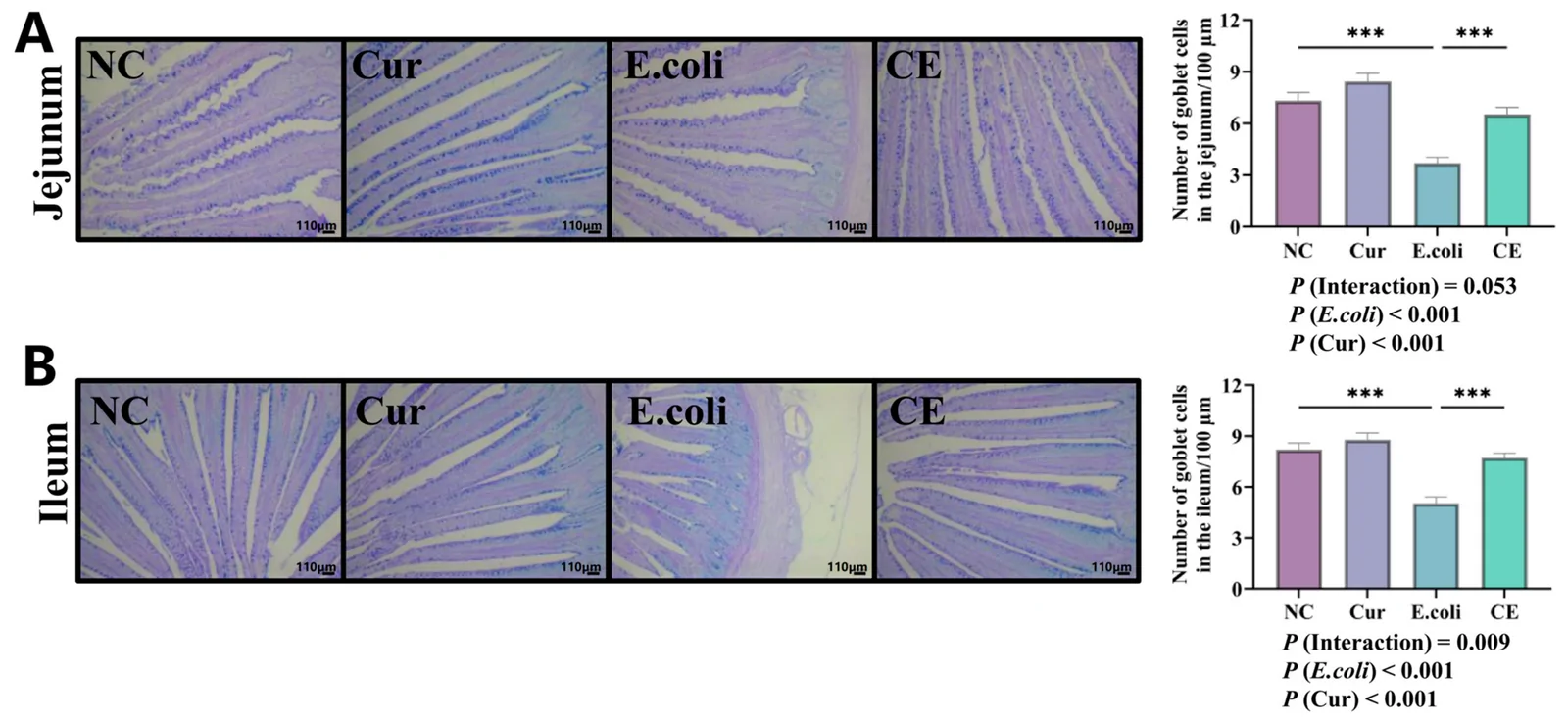
Curcumin restores goblet cell density in the jejunum and ileum of *E. coli*-challenged broilers. (**A**) Representative AB-PAS-stained sections and goblet cell density in the jejunum. (**B**) Representative AB-PAS-stained sections and goblet cell density in the ileum. *n* = 6 (6 broilers per replicate). Data are presented as mean ± SD. *** *p* < 0.001.

**Figure 4:**
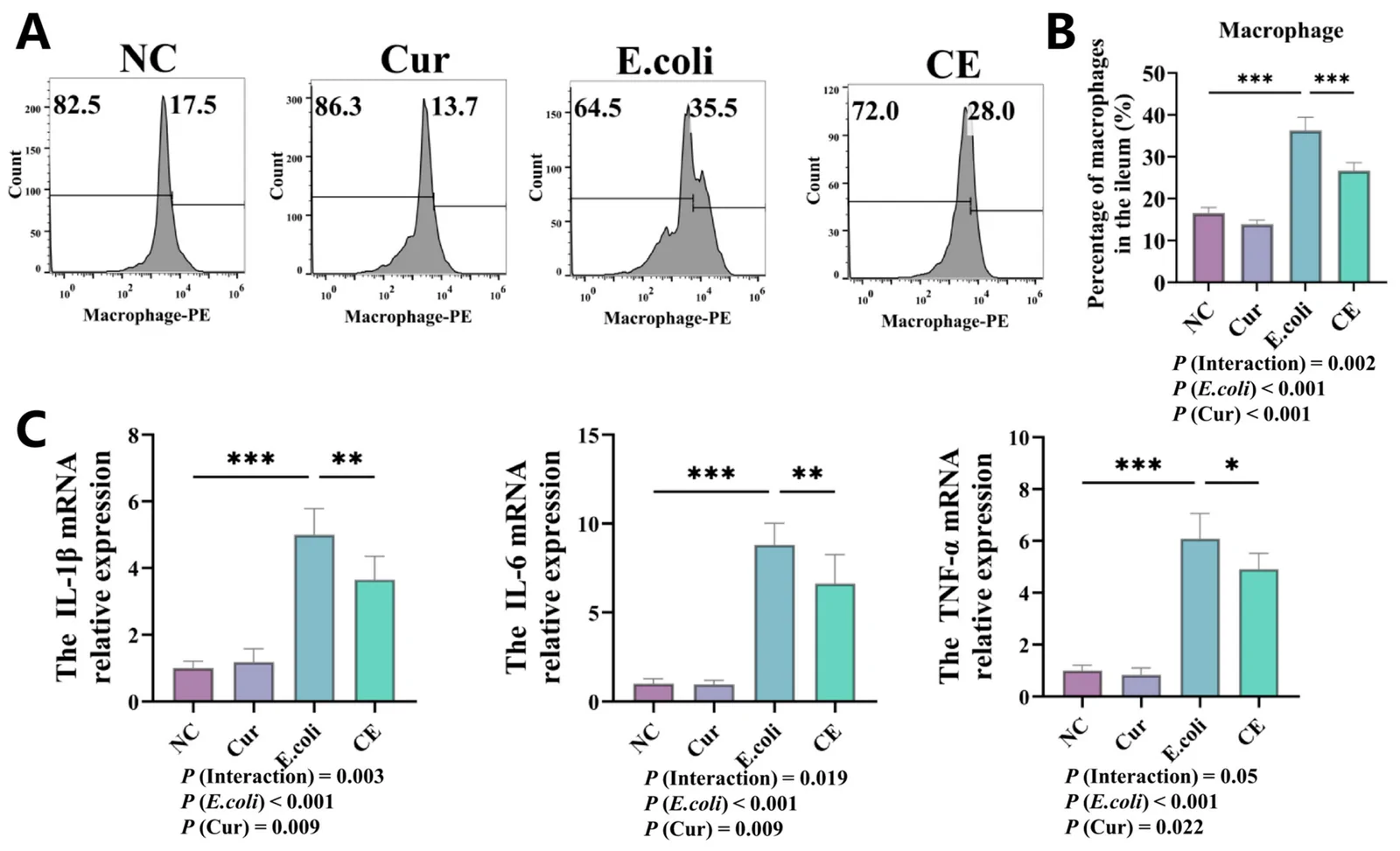
Curcumin suppresses ileal macrophage-mediated inflammatory responses in *E. coli*-challenged broilers. (**A**) Representative flow cytometry plots of ileal macrophages. (**B**) Percentage of ileal macrophages. (**C**) Relative mRNA expression of *IL-1β*, *IL-6*, and *TNF-α* in isolated ileal macrophages. *n* = 6 (6 broilers per replicate). Data are presented as mean ± SD. * *p* < 0.05, ** *p* < 0.01, and *** *p* < 0.001.

AB-PAS staining and flow cytometry were used to evaluate goblet cell density in the jejunum and ileum, as well as the number and functional status of ileal macrophages. Regarding goblet cells, the *E. coli* group showed significantly reduced goblet cell numbers (*p <* 0.001) in both the jejunum and ileum compared with the NC group, and goblet cell density was significantly increased in the CE group compared with the *E. coli* group (Figure 3A,B, *p <* 0.001), indicating that curcumin supplementation effectively restored the infection-induced loss of goblet cells. Flow cytometric analysis showed that the proportion of ileal macrophages (*p <* 0.001) was significantly decreased in the *E. coli* group compared with the NC group, whereas the proportion of macrophages was significantly increased in the CE group compared with the *E. coli* group (Figure 4A,B, *p <* 0.001). Meanwhile, the mRNA expression levels of pro-inflammatory cytokines, including IL-1β (*p <* 0.01), IL-6 (*p <* 0.01), and TNF-α (*p <* 0.05), in ileal macrophages were significantly lower (Figure 4C) in the CE group than in the *E. coli* group. These results suggest that curcumin not only increased the proportion of macrophages but also suppressed their inflammatory activation. Collectively, dietary curcumin supplementation alleviated *E. coli* challenge-induced intestinal barrier injury in broilers, in addition to increasing goblet cell density and modulating macrophage-mediated immune responses.

### 3.4. Curcumin Suppresses LPS-Induced Inflammatory Responses in HD11 by Inhibiting p38 MAPK Signaling

**Figure 5:**
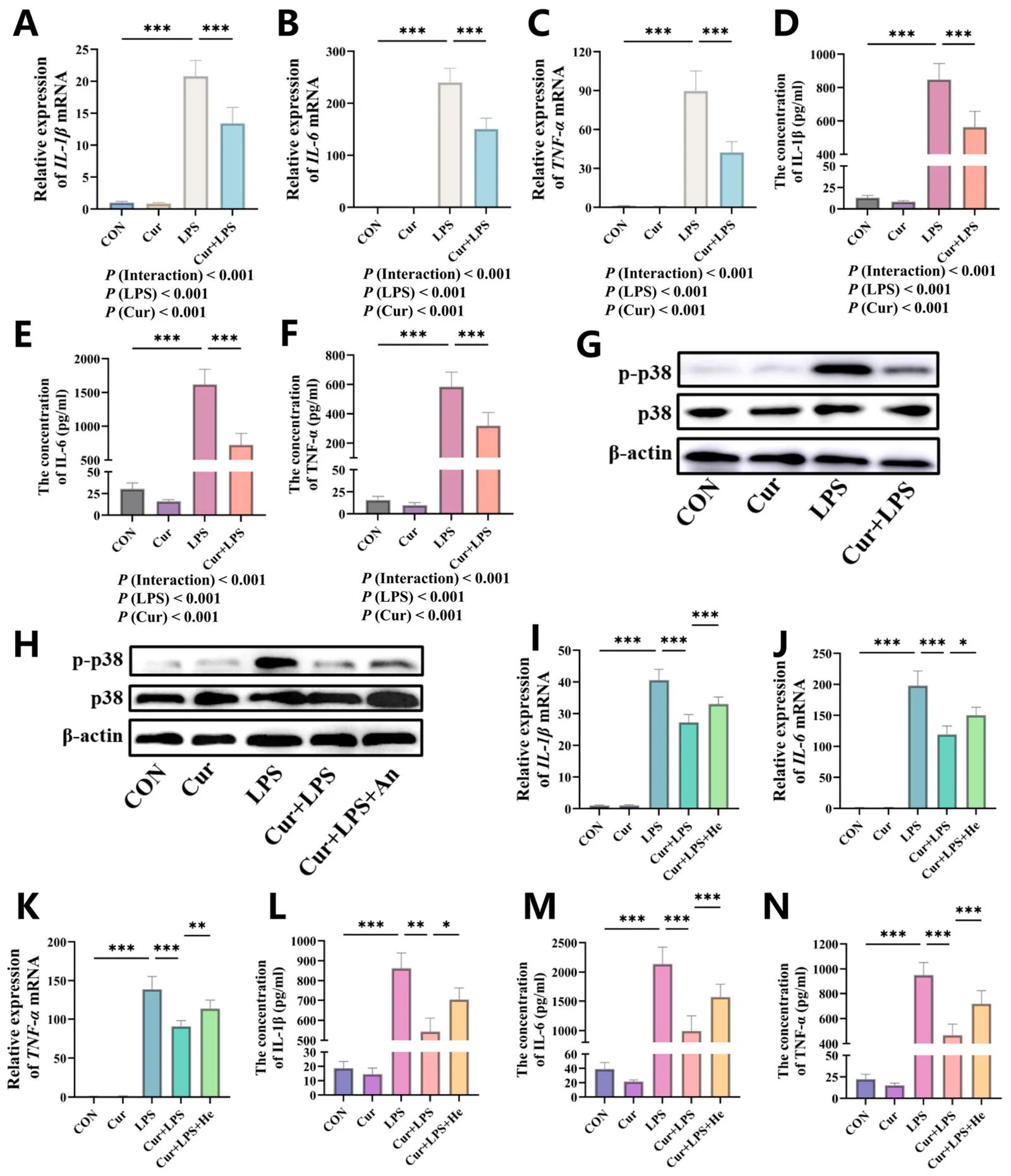
Curcumin suppresses LPS-induced inflammatory responses through inhibition of the p38 MAPK signaling pathway in HD11 cells. (**A**–**C**) Relative mRNA expression of *IL-1β*, *IL-6*, and *TNF-α*. (**D**–**F**) The concentrations of IL-1β, IL-6, and TNF-α in culture supernatants. (**G**,**H**) Representative Western blot images showing the levels of phosphorylated p38 (p-p38) and total p38. (**I**–**K**) Relative mRNA expression of *IL-1β*, *IL-6*, and *TNF-α* following anisomycin (An) treatment. (**L**–**N**) The concentrations of IL-1β, IL-6, and TNF-α following anisomycin treatment. *n* = 5. Data are presented as mean ± SD. * *p* < 0.05, ** *p* < 0.01, and *** *p* < 0.001.

Using chicken macrophage HD11 cells as an in vitro model, this study used LPS to simulate pathogenic stimulation and systematically evaluated the regulatory effect of curcumin on inflammatory responses, with further investigation of the potential mediating role of the p38 MAPK signaling pathway. The results showed that, at the gene expression level, LPS treatment significantly upregulated the mRNA expression of pro-inflammatory cytokines IL-1β (*p <* 0.001), IL-6 (*p <* 0.001), and TNF-α (*p <* 0.001). In contrast, co-treatment with Cur and LPS effectively reversed this effect and significantly decreased the expression levels of these inflammatory cytokines (Figure 5A–C, *p <* 0.01). At the protein level, LPS also significantly promoted the release of IL-1β (*p <* 0.001), IL-6 (*p <* 0.001), and TNF-α (*p <* 0.001), whereas Cur intervention markedly reduced their secretion (Figure 5D–F, *p <* 0.01). At the signaling pathway level, LPS stimulation significantly increased the level of p-p38 compared with the CON group, while curcumin co-treatment markedly inhibited p-p38 activation (Figure 5G). To verify whether curcumin exerted its anti-inflammatory effect through the p38 pathway, p38 activator anisomycin was added to the culture system. The results showed that the activator significantly reversed the inhibitory effect of curcumin on p38 phosphorylation, as indicated by the increased level of p-p38 in the LPS + Cur + An group compared with the LPS + Cur group (Figure 5H). Consistently, the mRNA expression and protein secretion of IL-1β (*p <* 0.05), IL-6 (*p <* 0.05) and TNF-α (*p <* 0.001) were also increased in the LPS + Cur + An group (Figure 5I–N). These findings indicate that curcumin exerts a negative regulatory effect on LPS-induced production of inflammatory cytokines in macrophages and that inhibition of p38 MAPK signaling contributes to the suppression of LPS-induced inflammatory responses in HD11 macrophages.

## 4. Discussion

The present study demonstrated that dietary curcumin supplementation effectively alleviated *E. coli*-induced enteritis in broilers by improving growth performance, preserving intestinal barrier integrity, and suppressing intestinal inflammatory responses. Moreover, curcumin attenuated LPS-induced macrophage inflammation through inhibition of the p38 MAPK signaling pathway, providing mechanistic evidence for its protective effect against intestinal inflammation.

Intestinal inflammatory cytokines serve as critical indicators of mucosal immune activation [19]. In this study, *E. coli* challenge significantly increased the levels of IL-1β, IL-6, and TNF-α in both the jejunum and ileum, indicating a robust local inflammatory response. These cytokines can amplify inflammation, exacerbate epithelial injury, and further compromise barrier integrity [20]. Curcumin supplementation markedly reduced the levels of these pro-inflammatory mediators, suggesting that curcumin effectively attenuates intestinal inflammation. This anti-inflammatory effect likely represents a key mechanism underlying the improvements in intestinal health and growth performance observed in curcumin-supplemented, challenged broilers.

Intestinal morphology reflects the digestive and absorptive capacity of the small intestine [21]. Here, *E. coli* challenge significantly reduced villus height and the villus height-to-crypt depth ratio in the jejunum and ileum, indicating structural damage and impaired absorptive function. The concurrent reductions in feed intake and body weight gain may be attributable to inflammation-driven mucosal injury and compromised nutrient absorption. *CLDN1*, *OCLN*, and *ZO-1* are essential tight junction components, whereas *MUC2* is critical for mucus layer formation [22]. The elevated expression of these genes in the curcumin-supplemented group indicates that curcumin enhanced both epithelial tight junction integrity and mucus barrier function, thereby reducing intestinal susceptibility to pathogen-induced injury. Goblet cells are responsible for mucus secretion and play a vital role in maintaining the intestinal mucosal barrier, pathogen exclusion and modulating host immune response [23]. Curcumin supplementation restored goblet cell numbers and upregulated *MUC2* expression, suggesting that it strengthens both the epithelial and mucus barriers. Together, these effects likely underpin the protective efficacy of curcumin against *E. coli*-induced intestinal injury.

In the present study, *E. coli* challenge significantly decreased the proportion of ileal macrophages, whereas dietary curcumin supplementation restored macrophage abundance compared with the *E. coli*-challenged group. More importantly, curcumin markedly reduced the mRNA expression of pro-inflammatory cytokines IL-1β, IL-6, and TNF-α in ileal macrophages, indicating that curcumin not only preserved the abundance of macrophages but also suppressed their inflammatory activation. Macrophages are key regulators of intestinal immune homeostasis and inflammatory responses. In addition to producing inflammatory mediators, activated macrophages participate in adaptive immune regulation by promoting T-cell activation and differentiation [24,25]. In particular, macrophage-derived IL-1β has been shown to promote Th17 cell differentiation, thereby amplifying intestinal inflammation [26,27]. Therefore, the reduced production of macrophage-derived inflammatory cytokines observed in the present study likely contributed to the attenuation of intestinal inflammation and the preservation of mucosal barrier function, suggesting that modulation of macrophage-mediated immune responses is an important mechanism underlying the protective effects of curcumin against *E. coli*-induced enteritis.

To further elucidate the molecular mechanism by which curcumin regulates macrophage inflammatory responses, HD11 cells were used as an in vitro macrophage model. LPS, a major component of the outer membrane of Gram-negative bacteria, activates Toll-like receptor 4 and subsequently triggers multiple downstream signaling pathways, including the MAPK pathway, thereby initiating inflammatory responses [28]. In the present study, curcumin markedly inhibited both the transcription and secretion of pro-inflammatory cytokines in LPS-stimulated HD11 cells, demonstrating a direct anti-inflammatory effect on chicken macrophages. Mechanistically, this inhibitory effect was accompanied by reduced phosphorylation of p38 MAPK, suggesting that suppression of p38 MAPK activation contributes to the anti-inflammatory activity of curcumin. As a member of the MAPK family, p38 MAPK plays a pivotal role in regulating inflammatory gene transcription in response to LPS stimulation [29]. Activation of p38 MAPK promotes the production of multiple inflammatory mediators, including TNF-α, IL-6, and IL-1β [30,31]. Previous studies have also demonstrated that several natural polyphenolic compounds, such as anthocyanins and catechins, exert anti-inflammatory effects by modulating the MAPK signaling pathway [32,33]. Consistent with these findings, pharmacological activation of p38 partially reversed the inhibitory effects of curcumin on inflammatory cytokine production in HD11 cells. These findings support the involvement of p38 MAPK inhibition in the anti-inflammatory effects of curcumin on chicken macrophages.

This study has several limitations. Intestinal colonization and bacterial load of the challenged *E. coli* were not directly quantified, which limited validation of the infection model. In addition, evidence for p38 MAPK involvement was derived mainly from LPS-stimulated HD11 cells and was not confirmed in intestinal tissues or isolated macrophages in vivo. Because the LPS model does not fully reproduce live bacterial infection or the intestinal microenvironment, contributions from other inflammatory pathways cannot be excluded. Moreover, only one curcumin dose, one *E. coli* strain, and one broiler genotype were examined, limiting assessment of dose–response effects and generalizability. Future studies should include quantitative infection validation, in vivo pathway confirmation, dose–response designs, and additional broiler models to clarify the mechanism and practical applicability of curcumin.

## 5. Conclusions

Dietary curcumin supplementation alleviated *E. coli*-induced enteritis in broilers by improving growth performance, reducing intestinal inflammation, preserving intestinal morphology, enhancing barrier function, and regulating macrophage-mediated immune responses, without significantly affecting the feed conversion ratio. In vitro experiments further suggested that inhibition of p38 MAPK signaling may contribute to the anti-inflammatory effects of curcumin on chicken macrophages.

## Data Availability

The datasets generated during the current study are available from the corresponding author upon reasonable request.

## Supplementary Materials

The following supporting information can be downloaded at: https://www.mdpi.com/article/10.3390/ani16182942/s1, Table S1: Ingredients and nutritional composition of the basal diet for chicks; Table S2: Primer sequences used for qPCR.

## Abbreviations

The following abbreviations are used in this manuscript:

AB-PAS	Alcian blue–periodic acid–Schiff staining
ADFI	Average daily feed intake
ADG	Average daily gain
CFU	Colony-Forming Unit
CLDN1	Claudin-1
*E. coli*	*Escherichia coli*
ELISA	Enzyme-Linked Immunosorbent Assay
FCR	Feed conversion ratio
H&E	Hematoxylin and eosin
IL-1β	Interleukin-1beta
IL-6	Interleukin-6
LPS	Lipopolysaccharide
MAPK	Mitogen-activated protein kinase
MUC2	Mucin 2
OCLN	Occludin
qPCR	Quantitative Real-Time PCR Analysis
SD	Standard deviation
TNF-α	Tumor necrosis factor- alpha
V/C	Villus height-to-crypt depth
ZO-1	Zonula Occludens-1
