# Supplementary material for: Curcumin Attenuates Escherichia coli-Induced Enteritis and Macrophage Inflammation in Broilers
Source: Animals (Basel). 2026 Sep 19;16(18):2942. doi: 10.3390/ani16182942 (PMC13603638; doi:10.3390/ani16182942)
Supplement: Supplementary file 1 [file animals-16-02942-s001.zip › animals-4478027-supplementary.pdf]

Table S1. Ingredients and nutritional composition of the basal diet for chicks

Component	Content, %	Nutrient composition[2], DM basis	Level, %
Corn	52.73	Metabolizable energy (MJ/kg)	12.38
Wheat	5	Crude protein	21.12
Soybean meal	33	Calcium	0.87
Corn gluten meal	3	Total phosphorus	0.65
Soybean oil	2.28	Non-phytate phosphorus	0.41
L-Lysine·HCl (98.5%)	0.06	Total Lys	1.1
DL-Methionine (99%)	0.1	Total Met	0.44
Limestone	1.05	Total Met+Cys	0.79
Dicalcium phosphate	1.48	Total Thr	0.74
Sodium chloride	0.3	Total Trp	0.21
Premix[1]	1	Total Ile	0.76
Total	100		

[1] The premix provided per kilogram of diet: vitamin A, 12,000 IU; vitamin D3, 600 IU; vitamin E, 45 mg; vitamin K3, 2.5 mg; vitamin B1, 2.4 mg; vitamin B2, 5.0 mg; vitamin B6, 2.8 mg; vitamin B12, 0.01 mg; choline, 1,300 mg; nicotinic acid, 42 mg; pantothenic acid, 12 mg; folic acid, 1.00 mg; biotin, 0.12 mg; Fe, 80 mg; Cu, 7.0 mg; Mn, 80 mg; Zn, 85 mg; I, 0.70 mg; Se, 0.15 mg.

[2] Total Lys, Met, Met+Cys, Thr, Trp, Ile, and ME were calculated values. Crude protein, total calcium, and total phosphorus were measured values.

Table S2 Primer sequences used for Q-PCR

Genes	Direction	Primer sequence (5′-3′)	NCBI References
OCLN	Forward	TCATCCTGCTCTGCCTCATCT	NM_205128.1
	Reverse	CATCCGCCACGTTCTTCAC	
ZO-1	Forward	CCAAAGACAGCAGGAGGAGA	XM_015278981.1
	Reverse	TGGCTAGTTTCTCTCGTGCA	
CLDN1	Forward	GAGGATGACCAGGTCAAGAAG	NM_001013611.2
	Reverse	TGCCCAGCCAATGAAGAG	
MUC2	Forward	CATTCAACGAGGAGAGCTGC	NM_204113.2
	Reverse	TTCCTTGCAGCAGGAACAAC	
IL-1β	Forward	TGCAGAAGGTAAGGATGGGAG	XM_046931582.1
	Reverse	TGGAAGGTGACGGGCTCAAA	
IL-6	Forward	CGATCCGGCAGATGGTGATAAA	NM_204628.2
	Reverse	GGAACAACACTGCCATCTGC	
TNF-α	Forward	CTGTGGGGCGTGCAGTG	NM_204267.2
	Reverse	GACAGGGTAGGGGTGAGGAT	
β-actin	Forward	GAGAAATTGTGCGTGACATCA	NM_205518
	Reverse	CCTGAACCTCTCATTGCCA	
